# AN ASSESSMENT OF THE COURT'S ROLE IN THE WITHDRAWAL OF CLINICALLY ASSISTED NUTRITION AND HYDRATION FROM PATIENTS IN THE PERMANENT VEGETATIVE STATE

**DOI:** 10.1093/medlaw/fwv026

**Published:** 2015-07-06

**Authors:** Simon Halliday, Adam Formby, Richard Cookson

**Affiliations:** 1 York Law School, University of York, Freeboys Lane, York YO10 5HD, UK; 2 Department of Sociology and Social Policy, University of Leeds, Leeds, UK; 3 Centre for Health Economics, University of York, York, UK

**Keywords:** Disorders of consciousness, PVS, Life-sustaining treatment, Best interests, Court of Protection, Declaratory relief

## Abstract

In this article, we reassess the court's role in the withdrawal of clinically assisted nutrition and hydration from patients in the permanent vegetative state (PVS), focussing on cases where health-care teams and families agree that such is in the patient's best interest. As well as including a doctrinal analysis, the reassessment draws on empirical data from the families of patients with prolonged disorders of consciousness, on economic data about the costs of the declaratory relief process to the National Health Service (NHS), and on comparative legal data about the comparable procedural requirements in other jurisdictions. We show that, following the decision in the *Bland* case, the role of the Court of Protection is now restricted to the direct supervision of the PVS diagnosis as a matter of proof. We argue that this is an inappropriate role for the court, and one that sits in some tension with the best interests of patients. The blanket requirement of declaratory relief for all cases is economically expensive for the NHS and thus deprives other NHS patients from health care. We demonstrate that many of the ancillary benefits currently offered by declaratory relief could be achieved by other means. Ultimately, we suggest that reform to the declaratory relief requirement is called for.

## I. INTRODUCTION^1^

According to Anthony Maden, ‘much of medical ethics consists of coming to terms with scientific discoveries.’^2^ The same might be said of medical law. The need to adapt to scientific and technological innovation is a charge often levelled at law in its relationship with medicine.^3^ What represents a therapeutic advance for medicine might, at the same time, pose a problem for law^4^—one that challenges the law to respond with its own innovations. An example of this, which forms the focus of this article, is the capacity of medicine to sustain the lives of patients in the permanent vegetative state (‘PVS’). Since the development of this diagnostic category in the early 1970s,^5^ legal systems across the world have had to grapple with a number of acutely difficult questions, the most challenging of which is whether the law should permit the continuation or termination of the treatments sustaining the life of such patients. In some jurisdictions, legal developments have been prompted or informed by high-profile and tragic cases,^6^ England and Wales amongst them. The situation of Anthony Bland, the young football fan catastrophically injured in the Hillsborough disaster, was the subject of the House of Lords' first consideration of an application to withdraw the medical treatment that was sustaining the life of a patient in the PVS.^7^ As is now well known, the court established a lasting precedent that it is not in such a patient's best interests to be maintained in life by clinically assisted nutrition and hydration (‘CANH’) and that it is lawful to discontinue it.

In the 20 or so years since the decision in *Bland*, the legal expectation in England and Wales has been that the court should be involved by way of declaratory proceedings in every decision to withdraw CANH from patients in the PVS, even where families and health-care teams agree that it is an appropriate course of action. The application for declaratory relief was originally framed in *Bland* as a matter of good practice. However, it is now a matter of legal obligation.^8^ A Practice Direction issued under the authority of the Court of Protection Rules 2007^9^ stipulates that:^10^

Cases involving any of the following decisions … should be brought to the court:

(a) decisions about the proposed withholding or withdrawal of artificial nutrition and hydration from a person in a permanent vegetative state or a minimally conscious state …

The termination of CANH of patients in the PVS can, of course, be a sensitive issue. It can be the source of distress and disagreement within families,^11^ between family members and doctors,^12^ or between doctors and other medical staff and carers.^13^ Equally, there are cases where medical experts cannot agree on the diagnosis.^14^ That such uncertainties and disputes should be resolved in the court is uncontroversial. However, the requirement that the court must consider all PVS cases, even in the absence of disputes or diagnostic disagreement, is not without controversy. British clinicians, it seems, are largely of the view that once a patient's vegetative state has been diagnosed as permanent, and where the family is in agreement, it should be possible to withdraw treatment without recourse to a court.^15^ Indeed, recent British Medical Association (BMA) guidance is explicit about this:

As expertise and professional guidelines develop on PVS, the BMA can see no reason to differentiate between decisions for patients in PVS and those for patients with other very serious conditions where ANH is not considered to be a benefit, which are currently governed by established practice without the need for legal review … The BMA hopes that in future the courts will decide that PVS cases no longer inevitably require court review, where consensus exists, as long as such withdrawal is in accordance with agreed guidelines.^16^

Professor Bryan Jennett (to whom responsibility for the diagnostic category is ascribed and who gave evidence in the Bland case)^17^ also questioned the continued blanket requirement of declaratory relief in the jurisdictions of the UK.^18^ Such is not required, he suggested, in any other jurisdiction outwith the UK.^19^

Legal scholars have also criticised the blanket requirement.^20^ Penney Lewis has queried the value of declaratory relief in uncontested cases in the light of its likely economic costs to the National Health Service (NHS) and the potential emotional costs to families. She has also questioned its rationality within the context of English medical law, pointing out that in other situations decisions to withdraw life-sustaining treatment do not require judicial approval:

What is special about PVS cases? Absent any judicial attempt to rationalize the special treatment of such cases, one can only speculate as to whether the rule requiring judicial approval in such cases reflects particular concern or is simply the result of historical accident … The judicial decision simply rubber-stamps the decision reached by the medical staff and the patient's family, having confirmed the diagnosis of PVS. The need to go court may cause distress to the patient's family and is expensive for the NHS.^21^

It is now over 40 years since the diagnostic category of PVS was established, and over 20 years since the English courts' first involvement in ‘best interests' decisions to withdraw CANH from patients in the PVS. Our aim in this article is to re-assess comprehensively the value of the English courts' continued involvement in cases where families and clinicians agree that the withdrawal is appropriate. A key task in an overall assessment of value, of course, is to identify the factors to be taken into account. Thus, we will consider the claims of Jennett and Lewis: (1) that English law is out of kilter with international legal trends, (2) that the requirement in relation to withdrawal of CANH from patients in the PVS is incoherent within the context of English medical law, (3) that it may be distressing for patients' families, and (4) that the procedure is economically expensive for the NHS. However, we must also consider contrasting claims that are made in favour of the requirement of declaratory relief. These we can find in the case law. The *Bland* case was explicit in setting out the justifications for the process. In the House of Lords, Lord Goff approved a statement of Sir Thomas Bingham MR in the Court of Appeal regarding the propriety of declaratory relief.^22^ Sir Thomas Bingham, in turn, was approving the ruling of Sir Stephen Brown P at first instance that declaratory relief should be sought in all cases:

This was in my respectful view a wise ruling, directed towards the protection of patients, the protection of doctors, the reassurance of patients' families and the reassurance of the public.^23^

This article proceeds by examining these contrasting claims about declaratory relief, exploring the extent to which they are merited. We then discuss these claims in relation to each other and come to an overall assessment about the value of the court's involvement in these cases. Ultimately, we argue that the blanket requirement for declaratory relief should be reformed.

## II. RESEARCH METHODS

Before moving on to examine the claims about declaratory relief, we must first consider the issue of research methods. As we can see, the claims made for and against the blanket declaratory relief requirement are of different types: some are legal and some empirical. And of the empirical claims, some are sociological and some economic. Here, we set out the research methods used to produce our various analyses.

To understand family responses to this issue, we draw on a series of publications from the Chronic Disorders of Consciousness Research Centre (of which we are a part) that have analysed in-depth narrative interviews with family members who have experience of having a relative in a prolonged disorder of consciousness, including the PVS. In addition to academic publications, there are summaries of key findings from this body of work, and film clips from some interviews, available online to the public as an educational resource at www.healthtalk.org. Of particular relevance to this article is the online page about ‘family experiences of applying to court for treatment withdrawal’, and we cite some of this online material where readers can hear and see film clips from the interviews for themselves.^24^

As regards our economic analysis,^25^ we draw heavily on previous NHS cost analyses for patients with severe and complex neurological disabilities based on the Needs and Provision Complexity Scale (NPCS) and the Northwick Park Care Needs Assessment.^26^ The NPCS is a tool for measuring the needs of patients with long-term neurological conditions and estimating the cost of meeting those needs. For our own ‘micro-costing’, we also obtained data on the unit costs of resource use from Unit Costs of Health and Social Care 2012.^27^ We have further made assumptions about particular cost parameters based on information from the recently published Royal College of Physicians' (RCP) best practice guidance for patients with prolonged disorders of consciousness^28^ and consultation with medical specialists with experience of the declaratory relief process. These specialists include two neurologists with experience as expert witnesses in PVS cases held at the Court of Protection. For the legal costs, standard legal administrative fees were identified from information published on the website of the Courts and Tribunals Judiciary. Published information on legal fees for barristers and solicitors and medical expert witness costs were not available, so we made assumptions based on discussions and correspondence with three barristers and two solicitors who have been involved in several PVS cases of declaratory relief.

Our comparative legal analysis is based in the first instance on a review of English language literature. We then supplemented this with data from ‘national rapporteurs' in additional jurisdictions. The use of national experts or rapporteurs to obtain legal data about foreign jurisdictions is an established research method in both legal^29^ and social policy^30^ research. It enables a level of descriptive comparison within a group of jurisdictions on a particular question of positive law. We contacted legal experts in a range of common law and European civil law jurisdictions. The responses we received are reported below.

## III. CLAIMS IN FAVOUR OF DECLARATORY RELIEF

In this section, we explore in more detail the claims in favour of the declaratory relief process even where families and health-care teams agree that CANH withdrawal is in the best interests of the patient.

### A. The Protection of Patients

The claim in the *Bland* case that declaratory relief would operate to protect patients referred to the protection of life. Declaratory relief would protect patients from unwarranted withdrawal decisions, knowing that withdrawal leads to death. Although, as a legal principle, the sanctity of life must sometimes yield to competing principles,^31^ it remains a fundamental element of medical law.^32^ Accordingly, given the relative novelty of the diagnostic category of PVS at the time and the ethical challenges associated with the withdrawal/withholding of life-sustaining treatments, the House of Lords in the *Bland* case judged that declaratory relief was an appropriate requirement of good medical practice—but only until a body of experience and practice had built up that would render the direct oversight of the courts unnecessary.

As we know, the question of whether it is in the best interests of a PVS patient to be kept alive through CANH is now settled as a matter of law.^33^ Equally, as a matter of formal medical knowledge, the diagnostic category of PVS is now well settled.^34^ However, in the context of the court's role in protecting patients' lives, we might still question whether diagnostic ‘method’ is settled as a matter of formal medical knowledge. Is there any critical debate within the medical community about appropriate diagnostic method and, if there is, does declaratory relief have any role in resolving it in order to be sufficiently confident in a particular PVS diagnosis? Equally, even if the issue of diagnostic method is settled, we might also question whether, as a matter of medical ‘practice’, it is observed sufficiently well to produce routinely reliable diagnoses. In other words, to what extent (if any) does the declaratory relief process offer greater confidence with respect to the PVS diagnosis?

#### 1. Diagnostic Method

At the heart of the standard process of diagnosing prolonged disorders of consciousness are the observation and assessment of patients' behaviour (or lack thereof).^35^ The extent of patients' awareness of self and environment is assessed by observations of how they respond (if at all) to various external stimuli. With the arrival of brain imaging technology, however, researchers have been able to observe more directly the functioning of the brains of patients in disorders of consciousness. The best known studies have used functional magnetic resonance imaging (fMRI) to analyse the brains of patients who have been asked to imagine different tasks (such as playing tennis). These have produced striking results. In a 2006 study,^36^ for example, the neural responses of a patient previously diagnosed as being in a vegetative state (though not a PVS) were found to be similar to a healthy volunteer. In a 2010 study,^37^ two patients who would otherwise have been considered as being in a vegetative state were found to be able wilfully to modulate brain activity. Such studies have led to considerable (albeit often exaggerated and inaccurate) media reports.^38^ They have also caused some medical researchers to question the quality of the behavioural method for diagnosing prolonged disorders of consciousness.^39^ Further, the Official Solicitor raised their significance during the declaratory proceedings of *B NHS Trust v J*,^40^ though the matter ultimately was not pursued.

This growing body of literature has subsequently been reviewed by a working party commissioned by the RCP. It noted that, although brain imaging should continue to be an important focus for research, the research has so far focussed on relatively small numbers of patients. It concluded that brain imaging has not yet reached a stage of development where it could be considered as part of routine clinical practice:

It remains unclear whether brain imaging is capable of informing the diagnosis beyond clinical and behavioural assessment and whether these techniques have any prognostic utility.^41^

For the time being at least, then, we can regard the matter of diagnostic method as being settled and authoritatively stated for England and Wales by the clinical guidelines of the RCP.^42^ Declaratory relief does not operate to protect patients in this respect.

#### 2. Diagnostic Practice

The question of the accuracy of routine diagnostic practice has also been the subject of a number of studies by medical researchers. In 1993, Childs et al.^43^ reported that 37% of patients (18 of 49) referred to a US neurorehabilitation unit had been incorrectly diagnosed as either comatose or in what was then termed a ‘persistent’ vegetative state. In 1996, Andrew *et al.* published a study^44^ that took place in a UK rehabilitation unit for those with profound brain damage, including the vegetative state. They reported that 43% (17 of 40) of patients referred to the unit with a referral diagnosis of vegetative state were subsequently found not to be such. Both of these studies are now relatively old. However, in 2009, Schnakers *et al.*^45^ reported, based on a study of patients in Belgium, that diagnostic ‘accuracy’ had not improved since the earlier studies, suggesting that 41% (18 out of 41) of patients had been inaccurately diagnosed as being in a vegetative state.

The claims of medical researchers that there is such a high rate of ‘misdiagnosis' have, understandably, proved rather alarming for medical lawyers.^46^ However, we must interpret these studies with care. The focus of the studies of both Childs *et al.* and Andrews *et al.* was on diagnoses of vegetative states, but not PVSs as we now know it. In the UK, vegetative states are now generally not deemed to be permanent until at least 12 months after a traumatic brain injury and 6 months after a non-traumatic brain injury.^47^ Yet, the Childs *et al.* study included patients whose brain injuries had not yet reached this timeline.^48^ Likewise, with the Andrews *et al.* study, as Wade^49^ has pointed out, 9 of their 16 ‘misdiagnosed’ patients on whom data could be presented^50^ were discovered to have communicative abilities before 12 months had elapsed since the onset of coma. In others words, it is possible that the cases in these studies were accurately diagnosed as ‘vegetative’ prior to referral, with patients' (not unexpected) emergence from the vegetative state occurring after referral. Equally, it is possible, as Childs *et al.* acknowledge, that diagnostic ‘inaccuracy’ is actually a result of confusion on the part of referring physicians about terminology.^51^

The study of Schnakers *et al.* fuses the issues of diagnostic practice and diagnostic method. Their claim about continued high rates of misdiagnosis is based on their finding that the use of a structured assessment tool as part of the diagnostic process produces different outcomes when compared with diagnoses that were based on a consensus amongst a medical team that arose from their daily (but unstructured) observations of patients. The key message from their research is that a structured assessment tool should be used in diagnosis in order to produce more reliable diagnoses. Yet, this is also the clear guidance from the RCP's recent clinical guidelines. Indeed, the RCP recommends the systematic application of at least two validated structured assessment tools before the diagnosis of PVS is made.^52^

The RCP's guidelines also note that the choice of assessment tool to be used will depend on the degree of ‘certainty’ needed for the decision in hand:

For example, assessment to support applications to the Court of Protection for withdrawal of clinically assisted nutrition and hydration (CANH) has critical impact on a serious and irrevocable decision. The Court will rightly expect a high level of certainty with respect to diagnosis.^53^

This reveals another reason for less alarm in the face of these research studies' headlines: the social reality of diagnosis is that it is an instrumental practice,^54^ in the sense that clinicians diagnose for particular purposes. Diagnosis for the purposes of referral to another medical unit, then, may not be of the same quality or confidence as diagnosis for the purposes of deciding whether to withdraw CANH. In other words, high rates of ‘misdiagnoses' at early stages of patients' disorders of consciousness will not necessarily translate into high rates of inappropriate withdrawals of CANH.

Nonetheless, diagnosis of the PVS is still a challenging and complex process. It should involve the interpretation of data from a range of sources, with the input of various health-care disciplines.^55^ The interpretive process that culminates in a diagnosis requires considerable expertise for it to be conducted properly.^56^ Furthermore, although there are good reasons to be cautious about the rates of ‘misdiagnosis' reported above, our discussions with experienced clinicians indicate that this diagnostic expertise for PVS is not widespread within the UK, perhaps due to the relative rarity of the disorder. Indeed, the number of clinical experts who have given diagnostic evidence in declaratory relief proceedings is very small indeed. The names that have appeared in recent case law can be counted in single figures, though the number of clinicians with the requisite expertise will, of course, be higher. But the challenges associated with diagnosis of PVS and the relatively small pool of expertise within the UK raise the question of whether a sufficient ‘body of practice’ (to frame it in the language of the *Bland* case) has built up to render the direct oversight of the courts unnecessary. The Official Solicitor always commissions an expert review of the diagnostic evidence as part of the declaratory relief proceedings. Usually, this supplements an expert review that the applicant (usually the NHS Trust) has already commissioned, sometimes using the same small pool of experts from which the Official Solicitor draws. It is clear, then, that the declaratory relief process has so far ensured considerable confidence in the PVS diagnoses prior to CANH being withheld or withdrawn. What is unclear, it is suggested, is whether the same quality and confidence of diagnosis would be achieved in every case, were it not for the declaratory relief process. It would certainly be possible for the guidelines of the various medical professional bodies to recommend a second-opinion procedure as they have done for CANH decision for patients who are not in the PVS (see further below). Equally, the RCP's recent clinical guidelines about diagnosing disorders of consciousness can guide and structure the diagnostic process. Nonetheless, until such times as the medical profession itself is confident of both the existence of widespread diagnostic expertise within England and Wales, and of routine adherence to the RCP's guidelines, we have to conclude that declaratory relief offers greater confidence in PVS diagnoses.

### B. The Protection of Doctors from Liability

The coroner responsible for dealing with the fatal cases of the Hillsborough disaster warned the doctor treating Anthony Bland (Dr Howe) that he may face criminal charges if he were to take action to bring Bland's life to an end. As Dr Howe has subsequently recorded:^57^

He made it clear that he ‘… could not countenance, condone, approve or give consent to any action or inaction which could be, or could be construed as being, designed or intended to shorten or terminate the life of this young man. This particularly applies to the withholding of the necessities of life, such as food and drink’. […] He sent copies of his letter to the Chief Constable […] and my defence society. On the following day I was visited at the hospital by a detective from Keighley Criminal Investigation Department (CID). He informed me that if I withdrew treatment and Tony died, I would be charged with murder.

It was on the basis of these warnings and further legal advice that Airedale NHS Trust sought declaratory relief in the *Bland* case. Given the legal uncertainty at the time, this was a prudent course of action on the Trust's part. As is well known, the court, in granting the declaration of lawfulness, was able to give Dr Howe and the NHS Trust the certainty they needed.^58^ But, of course, the *Bland* decision also set a precedent that should give certainty to other NHS Trusts faced with similar patients. It is now settled law that the withdrawal or withholding of CANH from patients in the PVS does not give rise to criminal liability for the consequent death of the patient.^59^ The legal cause of death is the event(s) that led to the vegetative state and not the withholding or withdrawal of CANH. The best interests justification for taking invasive steps to continue treating patients expires when the vegetative state becomes permanent. As the Supreme Court recently framed it on a related issue:

If the treatment is not in [the patient's] best interests, [i]t … follows that (provided of course that they have acted reasonably and without negligence) the clinical team will not be in breach of any duty towards the patient if they withhold or withdraw it.^60^

Declaratory relief, then, we can conclude, is no longer necessary for the protection of doctors from criminal or civil liability. Indeed, somewhat paradoxically, given that the point of substantive law is now settled, the requirement of declaratory relief probably gives rise to another form of liability: liability for contempt of court. The declaratory relief requirement is now a legal obligation that, by definition, introduces the possibility of non-compliance that would not otherwise be there.

### C. The Reassurance of Patients' Families

When reading case law, legal scholars are naturally drawn to the legal reasoning of a court's judgment. However, there is very little of this in the judgments relating to straightforward declaratory relief applications (where all parties are in agreement). As we noted above, in cases where the patient is deemed to be in the PVS, the court does not engage in the difficult ‘balance sheet’ deliberations that lead to a best interests determination. Instead, the focus of the Court of Protection in these cases is on the diagnosis as a matter of proof. The fact that there is very little legal reasoning in these cases should encourage us to observe what other functions these judgments might be performing. The reassurance of patients' families is one such function that continues to be performed, it is suggested. Although it is not an entirely uniform feature of this body of case law, with practices varying between particular judges, it is nonetheless common for judges in these cases to practice what some legal scholars have termed ‘therapeutic jurisprudence’.^61^ In other words, in deciding what to say in a judgment, judges clearly have in mind its emotional impact on the families of the patients concerned.

In the *Bland* case, Butler-Sloss LJ suggested that Anthony Bland existed in a ‘twilight world’.^62^ Lord Goff described his state as a ‘living death’.^63^ These descriptions of the PVS as neither fully life nor fully death accord with interview data with relatives of patients. For many, the ontological status of their relatives is not settled or clear.^64^ In this context, the judgments of the court in PVS cases take on a ceremonial function in formally moving the relative on from this state of limbo, from a twilight world towards full death. Indeed, in some important ways, the judgments often contain elements that one might commonly find in funerals or services of remembrance. For example, judgments often contain an element of eulogy whereby the patient is formally remembered. Some excerpts from recent cases illustrate this point:

CW … is described as being self contained, sensitive, quiet, shy and as having ‘so much depth of character and love’… CW cared for a number of pets and had a great love of animals. He enjoyed playing sport, rugby, hockey and cross country running at school and doing practical things such as helping his father with outdoor tasks, art, painting, stage scenery for school productions and, in particular, mechanical and engineering projects. He liked to take things apart and make things … His sister states that ‘CW was quiet but if you talked about engineering or mechanics you could not shut him up’.^65^

AW was a very active person. She ran a catering business. She was a keen sportswoman, who used to play squash and one year she ran the London marathon. She enjoyed music and dance … she will always be remembered as the unique and dynamic person that she was before she became so seriously unwell …^66^

It is also common for the family to be affirmed as being devoted to and loving of the patient:

Her devoted family have been assiduous in their attendance at the hospital and in their attempts to attain some sort of response from her. There have been many efforts to see if she had any ability to respond to those around her.^67^

JD's only surviving relative … is her cousin, who, along with his wife, also supports the application. They have been devotedly visiting JD regularly and frequently over the last two years.^68^

The loss to the family and the sadness of their situation are also commonly acknowledged:

the members of the family—not only those who have taken the trouble to come to court today—but others who are not here, and are feeling it just as much, I have no doubt, will be looking at the end of a life of a member of a family who was very dear to them.^69^

The last four and a half years have been very hard for AW's family, but their moving statements show that she is very much loved …^70^

And, finally, an assurance is often given that the death of the patient is the right course of action: indeed, that somehow medicine has interrupted the proper course of events. The underlying narrative here is that nature has determined that death must occur, and neither medicine nor law should stand in its way. The implication, it is suggested, is that no one—neither the family nor the medical team—need feel guilty or responsible:

It seems to me that no understanding of life is complete without an understanding of the proper place of death within that, and it is a human trait to desire what we commonly call ‘a good death' … I propose to grant the authorisation on the basis that artificial medicines should now withdraw and the natural order should be allowed to complete its course.^71^

The sympathies of this court go out to all of the family, friends, healthcare practitioners and carers who will be deeply affected by this decision. However, it is time for there to be peace.^72^

We can see, then, that although these judgments can be light on legal reasoning, they can contain a great deal of therapeutic jurisprudence. There are three main forms of what might be called ‘therapeutic benefits'. First, we can see that the declaratory relief judgments often operate as memorials of the living dead that anticipate the funerals that will follow in due course. The judgment of the court performs a function of ceremonially moving the family on from a state of limbo. Second, it also offers the family a formal affirmation of the extreme difficulty of their situation and the reality of their suffering. Emotionally, this can be significant. There is evidence of the comfort and reassurance that these judgments can give. For example, in one of the interviews for ‘healthtalk.org’, we are able to observe a relative's response to a declaratory relief judgment. Cathy was the sister of a PVS patient and is filmed reading the judgment for the first time (about 16 years after the event), having been presented with the transcript by the researcher. To fully appreciate the emotional impact of the judgment, one should watch the video.^73^ Nonetheless, the text of her words also conveys something of what she felt:

I do remember how kind everybody was at the court and how they talked a lot about the sort of level of care of Matthew and the devotion and the tragedy, which is what—[reads from court transcript] ‘it's a very moving account of how the family had to face the appalling tragedy which confronted them and how they had to live with the day-to-day knowledge which grew upon them that there would be no hope of recovery’. That's a good way of describing it: it's a knowledge that grows upon you. Last paragraph, he says: ‘I would like to express my appreciation of the tremendous care which has been given to M by his mother, father and sister and to express what I'm sure all those in court will wish to be associated with the very greatest admiration for their courage and the care which they've given to him [tearful]. I believe the time has come when the reality of the position must be recognised and accordingly, I make the declarations’ … I think it [the court judgment] is very well and almost rather beautifully expressed.

Cathy's statement also touches on the third way in which declaratory relief can be reassuring for relatives: as we saw above, the judgments often stress the appropriateness of letting the patient die. Research shows that family members often do not want to seek withdrawal of CANH. They hope instead that somehow ‘nature’ will take its course: for example, that death would come by way of an infection (albeit untreated).^74^ So, when a decision is finally made to permit death by withdrawal of CANH, the fact that this decision is made by the court can be significant. For such relatives, the jurisdiction of the court can help them not to feel responsible for the patient's death. It is important to recognise that, despite the difference in law between withdrawal of CANH and killing (which we discuss further below), these legalities mean little for relatives. For them, withdrawal of CANH is as much a way of ‘doing it’ (ie killing the patient) as would be a lethal injection. (Indeed, the prospect of withdrawing CANH is often deemed abhorrent, whereas lethal injection is often considered humane.)^75^ So, as our previous research has shown, for some the declaratory relief process shielded them from a sense of guilt:

… if the Court of Protection wasn't there to say, ‘Well *we* are making the ultimate decision and this is what *we* decide’, I would always feel that it was me who'd actually chosen to do it. And that would be hard … It would have been so hard to live with that, knowing that—almost feeling that you'd sentenced them to death, however much they wanted it. So the Court of Protection has shielded me from that experience, which is good.^76^

Although, as we note further below, the prospect of court hearings can be intimidating for some relatives, it is also clear that many of them are subsequently positive about their experiences. Declaratory relief, it seems, continues to operate as a reassurance for (at least some) patients' families.

### D. The Reassurance of the Public

Another of the benefits of the declaratory relief process suggested by the courts in the *Bland* case was the reassurance of the public. In the context of that case, this is understandable. It was the first UK case to consider the dilemma of whether to continue treating a PVS patient with CANH. It was reasonable, then, for the courts to speculate that their direct supervision of such medical decisions may reassure the public that appropriate decisions were being made. Nonetheless, any continued suggestion within the context of legal reasoning that the public needs reassurance on these issues (with the implication that declaratory relief can provide it) would be a legal fiction—an assertion of fact that operates for the purposes of legal reasoning but is not necessarily true.^77^ The reality is that we must remain agnostic about this.

Nonetheless, there is a reason to believe that there may be a continuing need for the reassurance of a significant minority of the general public. In the USA, between 2003 and 2005 when a similarly high-profile case (that of Terri Schiavo) was receiving sustained media attention, surveys suggested that between 27 and 42% of Americans were opposed to the withdrawal of CANH from the patient in the PVS, with 29% viewing CANH withdrawal as murder.^78^ Unfortunately, comparable data do not exist in relation to the UK, and it can be risky to ‘read across' too easily from another culture. However, we do know that some of the Parliamentary debates during the passage of the Mental Capacity Act 2005 featured concerns that withdrawal of CANH could amount to, or lead to, euthanasia.^79^ Equally, the joint committee of both Houses of Parliament reporting on the Bill recommended that the court always consider CANH decisions for patients in the PVS.^80^ It is, surely, reasonable to maintain the House of Lords' speculation in *Bland* that a not-insignificant proportion of the general public continue to be troubled by the prospect of allowing patients in the PVS to die.

Why might there be lingering discomfort for some sections of society about CANH withdrawal from patients in the PVS despite 20 years of legal clarity since *Bland*? Clearly, it relates (in part at least) to individual ethical convictions and/or religious doctrine^81^ on the issue. However, the more important point here, we suggest, is that such ethical standpoints may be underpinned by the difficulty of the legal reasoning in this field. What causes the death of the patient when CANH is withdrawn? Legally, the answer is the underlying brain injury that led to the PVS. But culturally, the answer is more likely to be the withdrawal of CANH itself. This is, no doubt, why in the case of *W v M*, the patient's sister felt able to express a preference for letting ‘nature take its course’ (by not treating infections) rather than CANH withdrawal.^82^ Legally, of course, the two courses of action are the same. It is also why family members often equate withdrawal of CANH with killing.^83^ One of the interviewees featured on ‘healthtalk.org’, for example, described her hopes that her mother would die ‘naturally’, without the need for CANH withdrawal:

I was probably hoping in the back of my mind, that she would take the initiative, she would have a heart attack and die. And that would be the resolution … I think it was mentioned that she might have—after we'd withdrawn the stroke—anti stroke tablets, my thought was, she'll die. She'll have another severe bleed or—I'm not even sure how it works. I thought she'd die. That's fine, that will happen and she will die, it'll be fine.

**Interviewer:** And her dying from a stroke or a heart attack would be better because?

It was her decision, not mine [laughs]. It was, you know, the body's saying. Not me having to say, make this blasted decision all the time. Her body giving up because it was just giving up, it had said, ‘I've had enough of life, I've had enough of living like this,’ that's it.^84^

It makes common (rather than legal) sense to perceive CANH withdrawal from PVS patients as the cause of death. Pointing to the difference between causation in law and causation in fact is unlikely to persuade anyone other than lawyers. As the RCP's recent clinical guidelines puts it:

The difficulty is that the longer a treatment is in place, the more it feels ‘normal’. Hence withdrawal of CANH may seem like a new or separate cause of a death…^85^

## IV. CLAIMS AGAINST DECLARATORY RELIEF

We can now examine in more detail the claims against a blanket requirement of declaratory relief, the first of which is that English law is out of kilter with the international trend.

### A. Is English Law Out of Kilter with the International Trend?

The specific question explored here is whether foreign jurisdictions required recourse to the courts in cases where (1) the prior wishes of the patient about treatment have not been formally recorded or established, and (2) the relevant parties (families, surrogate decision-makers, clinicians, etc.) agree that the withdrawal of CANH is the appropriate course of action. These conditions correspond to the usual situation encountered in England and Wales. Although the Mental Capacity Act 2005 provides both for the grant of surrogate decision-making power^86^ and for the legal effect of advance decisions,^87^ only a tiny proportion of the population of England and Wales have done either.^88^ In none of the reported cases were these applicable.

A fair amount has been written on this question. Jennet, for example, tells us that an international meeting in 1994 revealed that treatment withdrawal without recourse to the courts was accepted practice in Norway, Sweden, Denmark, and Japan.^89^ The same is true of New Zealand,^90^ the Netherlands,^91^ and South Africa.^92^ Similarly, in the USA^93^ and Australia,^94^ where the relevant jurisdictions are state level, court proceedings are not required. Northern Ireland, by way of contrast, however, follows England and Wales in requiring declaratory relief.^95^ On the basis of the national rapporteur data we received, we can also report that routine recourse to the courts before CANH can be withdrawn is similarly not required in the following jurisdictions: Belgium, Finland, France,^96^ Germany, and Portugal. In Ireland and India, however, the legal position appears to be the same as that of English law: declaratory relief from the courts must be sought, even where families and clinicians agree that withdrawal of CANH is appropriate.

Additionally, our national rapporteur data reveal that in (at least) two countries the withdrawal of CANH is not legally permitted. In Israel, it seems it is not permitted at all. In Italy, withdrawal of CANH is not permitted unless the patient has expressed in advance a wish to refuse medical treatment in that condition. Such a refusal would be most clearly expressed by way of a written advance decision, though a court, as a matter of proof, may conclude that an advance refusal of treatment has been made on the basis of evidence provided by family and friends.^97^

The legal position in Scotland is worth considering in a little more detail, particularly given its position as a sister jurisdiction within the UK. There is no equivalent in Scotland to the English Court of Protection Practice Direction requiring all cases to be brought before the courts. Indeed, the only reported case is *Law Hospital NHS Trust v Lord Advocate*,^98^ heard in 1996*.* Here, the court adopted a broadly similar position to the *Bland* case. Lord President Hope said:

I wish to emphasise that nothing in this opinion is intended to suggest that an application must be made to the court in every case where it is intended to withdraw treatment. The decision as to whether an application is necessary must rest in each case with those who will be responsible for carrying that intention into effect, having regard in particular to the views of the patient's relatives and any statements of policy which may, in the light of this case, be issued by the Lord Advocate.^99^

Policy guidance was, indeed, subsequently issued. Its most recent iteration states that:

… immunity from prosecution does not automatically extend to medical practitioners who have not sought and received the authority of the Court. The Lord Advocate has expressed the view, however, that if doctors and those acting on their instructions were acting in accordance with accepted medical practice and had exercised the proper degree of care expected of them, it would be very unlikely that any prosecution in the public interest would be brought against them.^100^

In light of this official guidance, there might legitimately be a suspicion that Scottish doctors do not feel obliged to seek declaratory relief from the court. However, for the purposes of this analysis, it would be better to regard the legal position in Scotland as a little uncertain.^101^ Accordingly, we may summarise our comparative findings by way of Table 1.

**Table 1.** Cross-jurisdictional comparison

Court decision required	Court decision not required	Withdrawal of CANH not permitted	Legal position unclear
England and Wales, Northern Ireland, Republic of Ireland, India	Australia, Belgium, Denmark, Finland, France, Germany, Japan, the Netherlands, New Zealand, Norway, Portugal, South Africa, Sweden, USA	Italy, Israel	Scotland

As shown in the table, we can see that Professor Jennett was not entirely accurate in suggesting that jurisdictions within the UK were unique as regards the requirement of declaratory relief. However, the broader point that Jennett was making is probably correct. Our data set is clearly not exhaustive of all foreign jurisdictions. Nonetheless, there appears to a fairly broad international trend to permit withdrawal of CANH without recourse to courts where families and health-care teams agree that it is appropriate to do. English law seems to inhabit a small pool of exceptions.

### B. Is the Requirement of Declaratory Relief Incoherent Within the Context of English Medical Law?

There are two ways of approaching this question. First, we may consider whether the obligation to obtain declaratory relief is coherent within the context of English law's general approach towards medical treatment. Second, we may then supplement this general discussion with comparisons of the procedural requirements relating to CANH and patients in the PVS with the procedural requirements in other relevant treatment situations. Specifically, we may compare the declaratory relief requirement with the general procedural demands regarding the withholding or withdrawal of CANH from patients who lack capacity [ie not confined to patients in the PVS or the minimally conscious state (‘MCS')]. We may also compare the declaratory relief requirement with the procedural demands regarding the withholding or withdrawal from patients in the PVS of life-sustaining treatment more generally (ie not confined to CANH).

#### 1. The General Legal Approach to Treatment Decision-Making

As is well known, a basic principle of English medical law is that patients may refuse medical treatment, even where the refusal is likely to be to their detriment.^102^ The basic logic of the law, then, is one of conservatism about the giving of treatment. There should not be treatment without consent.^103^ Thus, in relation to a course of treatment, when consent is withdrawn, treatment should be withdrawn. Likewise, where adults lack the capacity to give consent, the law maintains a conservative approach to treatment. As the Supreme Court noted in *Aintree University Hospitals NHS Foundation Trust v James*,^104^ ‘the focus is on whether it is in the patient's best interests to give the treatment, rather than on whether it is in his best interests to withhold or withdraw it.’^105^ As soon as a course of treatment is no longer in a patient's best interests, it ceases to be lawful to administer it.^106^ Given this essentially cautious approach to the lawfulness of administering medical treatment, we might question the coherence of requiring declaratory relief before CANH may be withdrawn from patients in the PVS where clinicians and families agree that it is an appropriate course of action. As we shall see further below, the requirement of obtaining declaratory relief usually causes a significant delay between the decision to withdraw CANH and its actual cessation. In this particular sense, then, declaratory relief, rather than protecting patients' interests, may actually be taken to be doing the opposite: it delays the treatment decision that is in the patient's best interests. Unlike cases where the patient has been diagnosed as being in the MCS, such delay is not required for the purposes of the judicial scrutiny of the best interests determination.^107^ The question of best interests has already been settled as a matter of law by the *Bland* case.

Delaying the actual withdrawal of CANH in such cases thus seems to sit in tension with the general thrust of the law here.^108^ The extent of this tension can be illustrated by comparing procedural requirements relating to CANH decisions for patients in the PVS with the procedural requirements in other relevant treatment situations.

#### 2. Comparing Withdrawal of CANH from Patients in the PVS with Other Withdrawal/ Withholding Decisions

The general position in English law is that, although good practice may require medical practitioners to seek declaratory relief where the legality of proposed treatment is in doubt, there is no general legal obligation to do so.^109^ As we can see from the *Bland* case, such was the position in relation to the withdrawal of CANH from patients in the PVS prior to the terms of the Code of Practice of the Mental Capacity Act 2005^110^ and the corresponding Court of Protection Rules.^111^ These provisions have thus made an exception to the general legal principle.^112^

Nonetheless, the question of good medical practice is also relevant here. How does the legal requirement of declaratory relief for withdrawal/withholding of CANH from patients in the PVS compare with good practice requirements surrounding CANH decisions more generally? In this respect too, it seems, the legal requirement is exceptional. As noted already above, the BMA sees no reason to differentiate between decisions for patients in the PVS and those for patients with other very serious conditions (other than MCS) where CANH is not considered to be a benefit, which are currently governed by established practice without the need for legal review.^113^ Equally, the guidance of the General Medical Council stipulates that, in relation to infants and neonates, CANH may be withheld or withdrawn where there is a consensus between the health-care team and the parents that it is not in the child's best interests. Likewise, in relation to adults who lack capacity (but who are not in the PVS or MCS) and are not expected to die within hours or days, CANH may be withheld or withdrawn where it is assessed not to be of overall benefit to the patient. In both contexts, however, a second opinion from a senior clinician who has experience of the patient's condition must be sought.^114^ Second opinions should, if possible, be based on an examination of the patient.^115^

In relation to patients in the PVS and life-sustaining treatments more generally, it is important to note that CANH is not the only life-sustaining treatment provided. Other life-sustaining treatments might include resuscitation in the event of cardiorespiratory arrest, or the use of antibiotics in the case of a life-threatening illness.^116^ It is not uncommon, for example, for patients in the PVS to develop pneumonia.^117^ Once again, we may note that there is no strict common law obligation for clinicians to seek declaratory relief in relation to such treatment decisions. For sure, in the *Bland* case, the courts stated that proposed withdrawal of life-sustaining treatments should be subject to declaratory proceedings. However, this was framed not as a matter of legal obligation, but as a matter of good practice—and only until a body of experience and practice had built up.^118^ This common law position, as we have already noted, has now been overridden by the Court of Protection Rules 2007.^119^ Yet, it seems to have overridden it only to the extent of decisions about CANH. It is worth reminding ourselves that the *Bland* case focussed on the lawfulness both of CANH and antibiotics treatment decisions.^120^ However, although it is not entirely clear,^121^ it seems that CANH decisions have been singled out as the only life-sustaining treatment decisions for patients in the PVS requiring court approval. Such is certainly the view of the RCP.^122^ Furthermore, the narrative within the case of *Re C*^123^ offers an example of where a decision to withhold antibiotics from a patient in the PVS was made without recourse to the courts. Research with family members confirms that this was not an isolated case.^124^ In respect of antibiotic treatment, it appears that the law has tacitly accepted that sufficient experience and practice have built up for decisions to be made, absent declaratory relief.

It seems, then, that patients in both the PVS and MCS have been singled out as requiring exceptional procedural protection in relation to CANH decision-making, despite the fact that, unlike MCS cases, the question of best interests for PVS patients has been settled as a matter of law. Equally, in relation to patients in the PVS, CANH has been singled out amongst other life-sustaining treatment decisions as requiring declaratory relief. As Lewis has suggested,^125^ there is no apparent legal rationale for this special treatment. Thus, the requirement of declaratory relief does not cohere well with broader English medical law.

### C. To What Extent Is the Declaratory Relief Process Distressing for Patients' Families?

Interview data certainly confirm that the declaratory relief process carries potential emotional burdens for families. First, for some relatives, the prospect of declaratory relief may be distressing because they feel that the court is an illegitimate forum for such decisions to be made.^126^ For some relatives, the requirement to go to court is an unwelcome legal intrusion into the family domain. Daisy, for example, wanted her brother to be permitted to die, but rejected the authority of the court to make that decision:

The decisions of the legal thing, that's what made me most angry … [T]here needs to be an option somehow. I mean, obviously you can't just be, ‘Hey he's [in a minimally conscious state], let's off him.’ But I don't think it should be a legal thing. I think it should be down to the doctors with the carers and it should be a multi-disciplinary thing. I don't think it *is* a legal matter.^127^

Although Daisy's brother was in the MCS, rather than PVS, we can presume that relatives of patients in the PVS could share such an attitude towards the court. Indeed, such seem to have been the Bland family's initial feelings about the prospect of court proceedings.^128^

Second, some relatives, like many lay people who face the prospect of involvement in court procedure, feel some anxiety about the formalities of the process. Lillian, for example, indicated that she ‘really didn't know what to expect’ of the court hearing. This caused her some anxiety, which was assuaged by a senior clinician with experience of the Court of Protection:

… at first I was thinking, ‘Oh my God, are they going to be interrogating me?’ … Professor [Jones] said, ‘No … it'd be highly unlikely they'd ask a member of the family to stand up and answer questions …’ […] He said, ‘you know, you don't have to be there’.^129^

Finally, the delay to the ending of the patient's life caused by the court process can be very upsetting for some families. Families with a relative in the PVS routinely experience grief, anger, exhaustion, and despair in relation to their relative's condition and treatment.^130^ Although it can take years for some families to come to a position of wanting their relative to be allowed to die,^131^ emotions around this desire can be intense once it exists. The considerable delay associated with the legal process that must currently follow the best interests decision to withdraw CANH is, for some, an additional and difficult burden. One family member said:

I still sort of question why it takes a year when three medical professionals are saying there's no hope. And then I realise it's got to go through the legal process … [T]hat will put another six months on it. So hopefully in … 18 months from starting we get the dignified end that we want as a family.^132^

However, the emotional impact of the declaratory relief process on families is by no means straightforward. Some relatives who discuss their negative perceptions of the legal process also express positive emotions about it. Lillian, quoted above, although being anxious about the formalities of court hearing, ultimately valued it. Another relative, despite his concerns about delay, still valued the sense of due process that declaratory relief offered:

These are momentous decisions that need to be done by a disinterested, authoritative and experienced party […] If you have a legal system, you can't have people taking the law into their own hands … I still support the fact you've got to have a system, you've got to stick to it.^133^

When assessing the emotional impact of the declaratory relief process, then, it is better to see this in complex rather than simple terms. Relatives may have mixed emotions that make it challenging to assess the legal requirement in this respect.

### D. What Are the Economic Costs to the NHS of the Declaratory Relief Process?

One of the core empirical realities underpinning health economics as a discipline is that limited budgets create difficult choices. In the context of a cash-limited NHS operating within a fixed budget, investment in one form of medical intervention comes at the cost of another. Accordingly, health economic analysis is often applied to guide treatment policy within the confines of health service provision. The economic cost of sustaining the lives of patients in the PVS and its implications for the provision of health services to other patients are clearly relevant factors to be considered when debating and assessing broader policy about prolonged disorders of consciousness. This is acknowledged both by members of the medical profession^134^ and the courts (though the courts indicate that such debate is the province of Parliament).^135^ We must stress, however, that our aim in this section is much narrower and less challenging: it is to assess the added costs to the NHS of the requirement of declaratory relief in cases where health-care teams and families agree that the withdrawal of CANH is appropriate.

The full details of our economic analysis and its assumptions can be found elsewhere.^136^ For the purposes of this article, we present only the main figures. We outline the additional costs incurred by the NHS ‘per case’ as a result of the declaratory relief requirement. It is not viable to estimate accurately the total savings to the NHS that legal reform would bring, as no reliable data exist about the number of patients in the PVS in England and Wales. (This is a problem that extends beyond these shores.^137^) Equally, it would be difficult to predict the overall effect of law reform on families' support for CANH withdrawal. Some families—those who are intimidated or offended by the prospect of legal process—may be more willing to support withdrawal of CANH if the need for declaratory relief were withdrawn. Yet, at the same time, others may be less willing. As we noted above, some families place value on the sense both of due process offered by court proceedings and of the court taking responsibility for the decision to allow the patient to die.

A number of assumptions underpin our economic analysis. The first is that the NHS funds the application for declaratory relief. A review of the case law reveals that this is usually (though not always) the case.^138^ The second assumption relates to the delay between a decision to withdraw CANH from a patient in the PVS and the declaration of that decision's lawfulness. Clearly, the actual time taken varies between cases. Only a few of the published judgments detail the timelines involved. Our economic analysis assumes that, on average, the delay is 9 months. This assumption derives from discussions with clinicians and lawyers who have considerable experience of the declaratory relief process. The calculation of the litigation costs also assumes conservatively that only two court hearings take place: one preliminary directions hearing and a final hearing. Of course, in some cases, things may initially seem straightforward but then later become contested: for example, if the expert medical witness commissioned by the Official Solicitor raises unexpected concerns about the PVS diagnosis requiring further medical assessment and a third hearing. Since this only happens in a small proportion of cases, however, this would not substantially increase our average cost estimates. So, to keep things simple and conservative, we do not explicitly cost out this scenario. As regards litigation, the largest item of cost to the NHS (constituting more than 80%) is the legal representation fees. This includes 100% of the fees for the barristers and solicitors acting for the applicant. We also include 50% of the fees for the barristers and solicitors acting for the Official Solicitor (who acts as the ‘litigation friend’ of the patient). By convention, these costs are also borne by the applicant.

**Figure 1:**
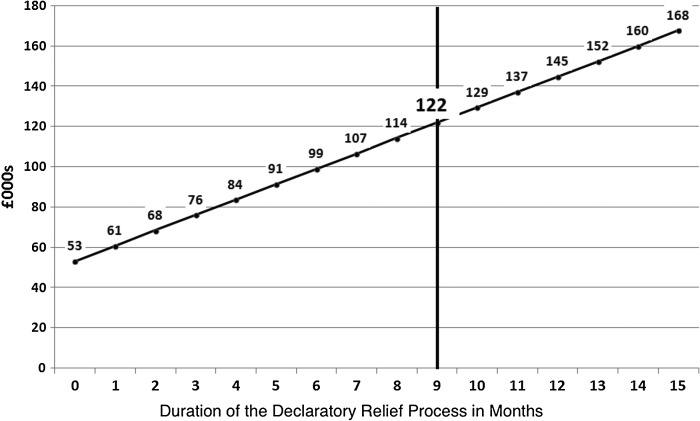
Average cost of the declaratory relief process to the NHS per case.

On the basis of these assumptions, we can estimate that the overall costs of the declaratory relief process to the NHS per case are, on average, about £122,000, comprising around £53,000 in litigation costs and £69,000 in ongoing care costs. Figure 1 offers a sense of the rising overall costs with the passage of time.

Given the relatively small numbers of applications for declaratory relief from patients in the PVS in England and Wales, the financial costs to the NHS are tiny relative to its overall budget. Although we have estimated that each case costs the NHS around £122,000, the overall yearly budget of the NHS is in excess of £105 billion.^139^ Yet, the NHS exists for the benefit of patients. Costs to the NHS are, ultimately, costs to individual patients in terms of foregone health care and the loss of health benefits from that foregone health care. It may be appropriate, then, to frame the costs of the declaratory relief process in terms of the standard measure of the benefit of health care used by the NHS: ‘QALYs'. The Quality-Adjusted Life Year is a measure devised by health economists for estimating the benefits produced by health interventions. It constitutes a common currency that enables explicit cost/benefit calculations across a wide range of health interventions. QALYs are calculated by estimating the years of life remaining for a patient following a particular treatment or intervention and weighting each year with a quality-of-life score (on a 0–1 scale).^140^ One QALY is equal to 1 year of life in full health. A year in less than full health will score less than 1. There is evidence that, on average, every £13,000 (approximately) of reduction in NHS expenditure causes the loss of at least one QALY.^141^ We can estimate, then, that each declaratory relief application (in straightforward cases) takes over 9 years of life (quality adjusted) from other patients.

## V. DISCUSSION

What should we make of these data? Our discussion of them proceeds in three stages. First, we can note that, in relation to straightforward cases where everyone is in agreement, the analyses above can be grouped into five main areas where the costs and gains of the declaratory relief process are felt: on patients, on families of patients, on the public more generally, on doctors, and on the law itself. These are the principal considerations that should populate a re-assessment of this question of law. Second, we can note that not all the benefits highlighted are necessarily tied to declaratory relief. Some could be maintained in other ways, even if the blanket requirement of declaratory relief were relaxed. Third, we must then consider the question of the ‘weight’ of the remaining considerations in relation to each other and how to drive legal policy in light on competing concerns.

### A. Five Main Considerations

As regards patients, the impact of the blanket declaratory relief requirement is somewhat mixed. With respect to patients with prolonged disorders of consciousness, there is good reason to believe that the blanket requirement operates to provide greater confidence in PVS diagnoses. This is significant, given the fact that the House of Lords in *Bland* settled the question of best interests for patients in the PVS, whilst in relation to patients in the MCS, the Court of Protection must determine each best interests question on its individual merits.^142^ The blanket declaratory relief requirement ensures in every PVS case both that the diagnostic guidelines of the RCP are complied with and that a third expert confirms the PVS diagnosis. It is not yet clear, it is suggested, that the quality and confidence of the PVS diagnosis would otherwise be as high in every case. Yet, we can also see that the blanket requirement operates to delay the course of action that is demanded by the best interests of most patients in the PVS. The cases where the declaratory relief process has led to an initial PVS diagnosis being revised to one of MCS are very few in number.^143^ For most patients in the PVS, then, the blanket declaratory relief requirement sits in some degree of tension with their best interests.^144^ At the same time, we have also estimated that each declaratory relief application takes over 9 years of life (quality adjusted) from other NHS patients.

As regards the families of patients, the evidence is also mixed. For some, the declaratory relief process will be a burden, either because the legal control over a relative's death is offensive, and/or because of the delay the process brings, and/or because of the anxiety that the legal process provokes—and some families may seek to avoid any such process with the result that CANH might continue without review, regardless of whether it is in the patient's best interests. For other families, the process will bring benefits, either because of the sense of due process being followed, and/or because of the authoritative reassurance it offers regarding the appropriateness of letting the relative die, particularly where it relieves them of a sense of guilt or responsibility. For others still, the declaratory relief process will be both benefit and burden.

As regards the public more generally, the evidence is, strictly speaking, insufficient. There is good reason to suspect that, despite (and because of) the tortuous legal reasoning in this field, many members of the public will equate withdrawal of CANH with euthanasia. And for many in society, this will be ethically unacceptable. But we simply do not know the extent to which the public needs reassurance about these decisions, and, perhaps more significantly, whether the declaratory relief process would provide it.

With respect to doctors, we have seen that the *Bland* case provided certainty for doctors that they would not face legal liability for CANH withdrawal from patients in the PVS. Indeed, the subsequent upgrading of the declaratory relief requirement from one of good practice to one of legal obligation under the Mental Capacity Act 2005 may well actually give rise to a risk of liability for contempt of court should the declaratory relief requirement not be complied with.

And finally, we should recognise that particular legal provisions may carry costs and benefits for the law itself in terms of its overall coherence. We have seen that English law is probably out of kilter with the international trend. This is not a cost, of course, but could operate as a benefit, should the law be changed. As the *Bland* decision demonstrates,^145^ domestic courts often engage in comparative scrutiny in the course of their deliberations and can take some comfort from it when aligning domestic law with an international trend.^146^ Nonetheless, we have also seen that the current obligation to obtain declaratory relief in all cases lacks a clear legal rationale in the context of domestic medical law. This must be considered to be a cost for the law.

### B. Benefits by Other Means

When systematically assessing the pros and cons of a particular legal obligation, there is a risk of overlooking the fact that some of the societal benefits offered by the legal obligation may be obtained by other means. Reform of the legal obligation thus may not necessitate the loss of all benefits. Which of the benefits outlined above may be maintained in other ways, should the blanket declaratory relief requirement be relaxed for ‘straightforward’ cases? Answering this question will help us focus on the central issues that must dominate this overall assessment of the declaratory relief requirement.

As regards the reassurance of families, we have seen that declaratory relief can have three main ‘therapeutic’ benefits: (1) it can have a ceremonial function in moving the family on from the unusual state of limbo, (2) it can affirm the extreme difficulty faced by families in such situations, and (3) it can reassure families about them not being responsible for the death of their relatives. Each of these functions could, it is suggested, be performed by carefully considered and formal best interests meetings between health-care teams and family members. The recent clinical guidelines of the RCP recommends that a formal best interests meeting takes place within 4 weeks of a diagnosis of PVS (ie once it is deemed ‘permanent’, which will be at least 6 months to a year after the original injury, depending on the nature of the injury). This meeting should involve the treating team, the family, and the responsible health authority, and its purpose should be ‘to decide whether continued life-sustaining treatment is in the patient's best interests’. It also recommends that families should be provided with support that is ‘practical, emotional and psychological’, noting ‘anticipatory bereavement work may be required’.^147^ Thoughtfully planned best interests meetings thus could render the ‘therapeutic justice’ work of the courts unnecessary.

As regards the reassurance of the public, we have seen that the House of Lords in *Bland* supported the notion that declaratory relief would help achieve this. Yet, we have also seen that, because PVS is a chronic state of limbo—being ‘permanently’ caught between life and death—culturally, withdrawal of CANH ‘feels’ like medical euthanasia. The unusual and distinctive blanket necessity of declaratory relief can only add to this cultural sense. There is thus a paradox in the fact that the attempts of the court to give public reassurance may now actually (in some ways at least) achieve the opposite: in terms of substantive law, CANH withdrawal from patients in the PVS is no different from other treatment situations, and the cause of the death is the pre-existing injury; in terms of procedural law, however, it is very different and has elevated significance in requiring prior court approval. To this extent, the law is giving mixed messages. If public reassurance is a legitimate aim of the law here, surely it is now time to offer that reassurance by not treating CANH decisions for patients in the PVS as qualitatively different? Such equal treatment would serve to emphasise the applicability to such decisions of the ordinary principles and practices of medical law: that, in the absence of disagreements, doctors will withdraw treatment as soon as it is no longer in the patient's best interest.

At the same time, when considering the issue of public reassurance, we should not ignore members of society who have opposing views to those noted above. For this section of society, reassurance will relate to the upholding of human dignity in allowing patients in the PVS to die as soon as continued life is not in their best interests. It is worth recalling that both sets of core values were alluded to in the judgment by Hoffman LJ in the Court of Appeal in the *Bland* case:

In my view the choice the law makes must reassure people that the courts do have full respect for life, but that they do not pursue the principle to the point at which it has become almost empty of any real content and when it involves the sacrifice of other important values such as human dignity and freedom of choice.^148^

Ultimately, public reassurance, should it come, may be better emanating from the legislature rather than the court. Indeed, as we note further below, this would be a requirement of legal reform in any event.

With respect to protecting doctors from legal liability, we have seen that, in the main, this has been achieved through the terms of the substantive law set out in *Bland*. Reform of the blanket requirement of declaratory relief would not alter this. As regards the protection of patients, could the level of diagnostic confidence currently ensured by declaratory relief be achieved without it? As we saw above, the answer to this question is ‘probably not’—at least for the moment. Thus, we arrive at the one benefit that currently is most clearly guaranteed by way of declaratory relief alone. It is also the most significant benefit promised by it, we suggest. For these reasons, it must take the central place in the discussion of whether the law should be reformed. It is to this issue that we now turn.

### C. How to Resolve Competing Considerations

The industry of law, including legal scholarship, is fond of the metaphor of ‘balance’.^149^ Indeed, even within the narrow field of law being focussed on here, we know that in MCS cases the Court of Protection must perform the impossible task of a ‘balance sheet’ exercise when deciding whether it is in a patient's best interest to withdraw CANH.^150^ The important point here is that the task really is impossible. The metaphor of ‘balance’ is evocative, and may be reassuring, but is ultimately false.^151^ It suggests that competing considerations are commensurable. But most complex problems in law are such precisely because the issues cannot easily be measured against each other.

This, of course, is the problem we have here. How, for example, do we judge the ‘weight’ of greater confidence in PVS diagnoses against the (quality-adjusted) life years for other NHS patients? Or against the delays in implementing best interests decisions of many patients in the PVS? Or against the potential distress of families having to negotiate the declaratory relief process? In the absence of a common unit of measurement, we must find another approach to the resolution of our central question. Our suggestion is that we can gain sufficient clarity on this issue by ‘zooming out’ from it for a moment and thinking about the role of medical law in its broader socio-legal context.

The pragmatic and empirical context within which the principles of medical law must do their work is the functioning of the NHS. The NHS budget (noted above) is astronomically high because it treats over one million patients every 36 h.^152^ Medical law is one of the ways (and a symbolically powerful one at that) by which the routine work of the NHS is regulated. The regulatory effectiveness of medical law is achieved, in the main, by the internalisation of legal values and principles as professional values and principles, with only the very occasional intervention of litigation and the courts. The vast majority of treatment decisions (including those for patients without capacity and for the end of life) are made in the absence of direct legal involvement. This, of course, is necessary for the efficient operation of the NHS given the size of its workload. Efficiency of public administration is itself a (sometimes undervalued) legal value.^153^

However, there is a tiny minority of unusually demanding medical situations where the law reserves decision-making for the courts alone. For the purposes of this analysis, we can note that, amongst others, the law has reserved decisions about whether to treat patients in the MCS and PVS with CANH for the exclusive attention of the court. This was understandable because the question of whether such treatment is in the best interests of a patient is very challenging, given that these patients lack the capacity to make treatment decisions themselves and that withdrawal of CANH will lead to death. Courts are well suited to the unenviable task of resolving such difficult and consequential individual best interest decisions.^154^ For patients in the MCS, each case is different and must be considered on its merits. For patients in the PVS, however, the House of Lords, in deciding what was in the best interests of Anthony Bland, determined it for all subsequent patients too. In that respect, all PVS cases are now the same. So, the Court of Protection is no longer routinely involved in best interests decisions for patients in the PVS. Instead, its role is now restricted to confirming diagnoses as a matter of proof.^155^

Can this be justified? The courts have certainly long had a role in resolving claims of negligent diagnosis. But that is not what is happening here. In relation to PVS and intended CANH decisions, the court is actually supervising the diagnosis before a treatment decision can be put into effect. Although it may have been appropriate that the court was called on in *Bland* to resolve the particularly challenging best interests decision, it is hard to think of a principled basis (outwith situations of dispute or disagreement) for the courts' continued involvement in PVS diagnostic decisions, now that the best interest matter is settled. In the grand division of labour, medical diagnosis falls appropriately within the province of the health system and not the courts.

Nonetheless, we might still ask whether there is anything distinctive about PVS as a diagnostic category that justifies the unusual and direct judicial supervision of individual diagnoses. The answer to this question is clearly and obviously ‘no’. It would be strange to suggest that the court must supervise all PVS diagnoses. Rather, recalling that diagnosis is an instrumental business, the better question is whether there are specific and distinctive purposes for which PVS diagnoses are made that call for direct judicial supervision of the diagnosis. To put the matter more concretely, is the purpose of letting a patient die sufficiently distinctive and unusual that it calls for direct judicial supervision of the diagnosis itself? Again, we suggest the answer is ‘no’. The truth is that end-of-life treatment withdrawal/withholding on the basis of medical diagnosis is routine within the NHS.^156^ Indeed, as we saw above, the Mental Capacity Act 2005 and its associated Code of Practice and Rules of Court appear to have tacitly accepted that doctors may make judicially unsupervised best interests decisions to withhold antibiotics or cardiopulmonary resuscitation from patients in the PVS on the basis of judicially unsupervised diagnoses. The current requirement for the PVS diagnosis to be confirmed in court before CANH may be withheld or withdrawn is truly anomalous.

## VI. CONCLUSION

In this article, we have argued that it is time for the blanket declaratory relief requirement before CANH may be withheld or withdrawn from patients in the PVS to be reformed. Yet, at the same time, we have recognised that this is a sensitive policy issue and that it is likely that some level of public reassurance would be prudent (on both sides of the debate). From whom should such reassurance come? We are, unfortunately, at risk of being caught in law reform stalemate. When the issue of declaratory relief was debated during the passage of the Mental Capacity Bill, the government indicated that it preferred to wait for the courts to take the reform initiative:

Case law has set out … categories of case that should have prior sanction of the court [including] the discontinuance of artificial nutrition and hydration for a patient in a permanent vegetative state … The Government intend the situation … to continue under the Bill. The current law has developed by guidance in case law responding to difficult cases. We want the courts to continue to decide which cases should have their prior sanction […] For that reason, we consider that it would be most effective to use the codes of practice to specify the situations where decisions should be taken only by a court.^157^

This was, perhaps, an important and skilful way of securing a successful conclusion to a very long-running and complex legislative process.^158^ However, it is something of a fudge. The higher courts may, of course, give a signal that procedures should change, but it is not necessary before reform can take place. Indeed, such a change would formally have to come from the legislature in any event. The Court of Protection Rules^159^ have been made under the power of the Mental Capacity Act 2005^160^ and so have the authority of statute. Equally, although the Lord Chancellor is empowered to make revisions to the Code of Practice, a new code would have to be laid before both Houses of Parliament.^161^ It is hoped that in due course there will be the opportunity to do so in relation to cases where families and health-care teams agree that withdrawal of CANH is in the PVS patient's best interests.

## Footnotes

1 We are very grateful to Penney Lewis and Lynne Turner-Stokes who read and commented on earlier drafts. The analysis also gained much from the feedback of York Law School colleagues in its ‘Brown Bag’ internal seminar series. We are also very grateful to a number of national experts who helped us with the law in other jurisdictions: Kathy Cerminara (USA), Stefano Civitarese and Alessandra Pioggia (Italy), Ruth Horn (France and Germany); Mary Donnelly (Ireland), Ben White and Lindy Willmot (Australia), Herman Nys (Belgium), Andre Dias Pereira (Portugal), Melanie Slabbert (South Africa), Sirpa Soini (Finland), Avraham Steinberg (Israel), David Stephenson (Scotland), and S.V. Joga Rao (India). Finally, we are grateful to our colleagues in the Chronic Disorders of Consciousness Research Centre who commented on this work while in progress, particularly Derick Wade, Celia Kitzinger, and Jenny Kitzinger.

2 A Madon, ‘England's New Mental Health Act Represents Law Catching Up with Science: A Commentary on Peter Lepping's Ethical Analysis of the New Mental Health Legislation in England and Wales' (2007) 2 Philosophy, Ethics and Humanities in Medicine 16.

3 MH Shapiro, ‘Is Bioethics Broke?: On the Idea of Ethics and Law “Catching up” with Technology’ (1999) 33 Ind. L. Rev. 17; L Bennett Moses, ‘Understanding Legal Responses to Technological Change: The Example of In Vitro Fertilization’ (2005) 6 MJLST 505; L Bennett Moses, ‘Recurring Dilemmas: The Law's Race to Keep Up with Technological Change’ (2007) 7 U. Ill. J.L. Tech. & Pol'y 239.

4 S Harmon, ‘Consent and Conflict in Medico-Legal Decision-Making at the End of Life: A Critical Issue in the Canadian Context’ (2010) 60 U. N. Brunswick L. J. 208.

5 B Jennett and F Plum, ‘Persistent Vegetative State After Brain Damage: A Syndrome in Search of a Name’ (1972) 1 Lancet 7753.

6 In the USA, for example, the cases of Quinlan (*In the Matter of Karen Quinlan, an Alleged Incompetent* 70 N.J. 10 (1976)) and Schiavo (*Schindler v Schiavo* 780 So 2d 176, 177 (Fla Dist Ct App.), 2001)); in India, the case of Shanbaug (*Aruna Ramchandra Shanbaug v Union Of India* (2011) 4 SCC 454); and in Italy, the case of Englaro (Cassazione—Sentenza N° 21748/07 (Caso Englaro)).

7 *Airedale NHS Trust v Bland* [1993] AC 789.

8 *W v M and others* [2011] EWHC 2443 (Fam).

9 S.I. 2007/1744.

10 Practice Direction 9E, paragraph 5. Except for the additional mention of the minimally conscious state, this mirrors the terms of the Mental Capacity Act 2005′s Code of Practice (sections 6.18 and 8.18).

11 For example, *Re G* [1995] 2 FCR 46.

12 For example, *NHS Trust v D* [2005] EWHC 2439.

13 For example, *Re C* [2010] EWHC 3448 (Fam); *Re J* [2012] All ER (D) 146 (Nov.).

14 For example, *Re D (Medical Treatment)* [1998] 2 FLR 36; *NHS Trust A v H* [2001] 2 FLR 501.

15 A Grubb and others, ‘Survey of British Clinicians’ Views on Management of Patients in Persistent Vegetative State’ (1996) 248 Lancet 35.

16 British Medical Association, *Withholding and Withdrawing Life-Prolonging Medical Treatment: Guidance for Decision Making* (Blackwell Publishing: Oxford, 3rd edn, 2007), 61.

17 Jennett and Plum (n 5) 734. Jennet, however, notes that he was not the first to report the condition that has come to be known as PVS. See B Jennett, ‘A Quarter Century of the Vegetative State: An International Perspective’ (1997) 12 J. Head Trauma Rehabil. 1.

18 B Jennet ‘Should Cases of Permanent Vegetative State Still Go to Court?’ (1999) 319 BMJ 796.

19 Jennett (n 17).

20 See R Huxtable, ‘“In a Twilight World”? Judging the Value of Life for a Minimally Conscious Patient’ (2014) 39 J. Med. Ethics 565.

21 P Lewis ‘Withdrawal of Treatment from a Patient in a Permanent Vegetative State: Judicial Involvement and Innovative “Treatment”’ (2007) 15 Med. L. Rev. 392, 396, and 397.

22 *Airedale NHS Trust v Bland* [1993] AC 789, per Lord Goff at 873.

23 Ibid, 815.

24 For a full description of the methods and sample for this research, see the publications referenced: n 54, n 64, n 76, n 117, and n 130. Although it is not actually known how many cases of declaratory relief there have been in England and Wales, the sample of families represented within this body of work is likely to be fairly small in relative terms—5–10% of the whole depending on which estimate of the whole is assumed to be correct. In 2004, the government reported figures in response to a Parliamentary question (HC Deb 09 February 2004, vol. 417, c1208W). In the 11 years since the first case (1993–2004), there had been 36 applications for declaratory relief (including the *Bland* case). Eight cases have been reported since then. However, many judgments in the Court of Protection are never reported, and we know that some decisions about CANH withdrawal from PVS patients have been among them. It has recently been estimated that there have been more than 100 cases since *Bland*: C Kitzinger and J Kitzinger, ‘Family Perspectives on Proper Medical Treatment’ in S Fovargue and A Mullock (eds), *The Legitimacy of Medical Treatment: What Role for the Medical Exception*? (Taylor and Francis: London 2015, in press).

25 A full description of our costing methods is provided in A Formby, R Cookson, and S Halliday, *Cost Analysis of the Legal Declaratory Relief Requirement for Withdrawing Clinically Assisted Nutrition and Hydration (CANH) from Patients in the Permanent Vegetative State (PVS) in England and Wales* (Research Paper 108, Centre for Health Economics, University of York, 2015).

26 L Turner-Stokes, K Nyein, and D Halliwell, ‘Care Needs Assessment—A Directly Costable Outcome Measure for Rehabilitation’ (1999) 13 Clin. Rehabil. 253; L Turner-Stokes, A Bill, and R Dredge, ‘A Cost Analysis of Specialist Inpatient Neurorehabilitation Services in the UK’ (2012) 26 Clin. Rehabil. 256; L Turner-Stokes, P McCrone, DM Jackson, and RJ Siegert, ‘The Needs and Provision Complexity Scale: A Multicentre Prospective Cohort Analysis of Met and Unmet Needs and Their Cost Implications for Patients with Complex Neurological Disability’ (2013) 3 BMJ Open, doi:10.1136/bmjopen-2012-002353.

27 LA Curtis, ‘Unit Costs of Health and Social Care 2012’ (Personal Social Services Research Unit, 2012) <http://www.pssru.ac.uk/project-pages/unit-costs/2012/> (last accessed 29 January 2015).

28 Royal College of Physicians, *Prolonged Disorders of Consciousness: National Clinical Guidelines*(RCP: London 2013).

29 See, eg, A Böcker and E Guild, *Implementation of the Europe Agreements in France, Germany, the Netherlands and the UK: Movement of Persons* (Platinium Publishing Limited: London 2002); VV Palmer, ‘From Lerotholi to Lando: Some Examples of Comparative Law Methodology’ (2005) 53 Am. J. Comp. L. 261; BJ Rodger, ‘Art 234 and Competition Law: A Comparative Analysis' (2008) 15 Masst. J. Eur. & Comp. L. 149.

30 See, for example, M Adler and P Henman, ‘Computerizing the Welfare State’ (2005) 8 Inform. Commun. Soc. 315; C Skinner and J Davidson, ‘Recent Trends in Child Maintenance Schemes in 14 Countries' (2009) 23 IJLPF 25.

31 *Airedale NHS Trust v Bland* [1993] AC 789.

32 *W v M and others* [2011] EWHC 2443 (Fam).

33 *Airedale NHS Trust v Bland* [1993] AC 789.

34 RCP (n 28).

35 Ibid.

36 AM Owen and others, ‘Detecting Awareness in the Vegetative State’ (2006) 313 Science 1402.

37 MM Monti and others, ‘Willful Modulation of Brain Activity in Disorders of Consciousness' (2010) 362 N. Engl. J. Med. 579.

38 G Samuel and J Kitzinger, ‘Reporting Consciousness in Coma: Media Framing of Neuro-scientific Research, Hope, and the Response of Families with Relatives in Vegetative and Minimally Conscious States' (2013) 3 JOMEC J. 1.

39 See, for example, J Giacino and others, ‘Behavioral Assessments in Patients with Disorders of Consciousness: Gold Standard or Fool's Gold?’ (2009) 177 Prog. Brain Res. 33.

40 [2006] EWHC 3152 (Fam). After consultation with an expert witness, the Official Solicitor ultimately concluded that it would not be in the interests of *J* to undergo an fMRI scan.

41 RCP (n 28) 28.

42 Ibid. These guidelines have been endorsed by the following bodies: the Association of British Neurologists, the Association for Palliative Medicine, the British Society of Rehabilitation Medicine, the Chartered Society of Physiotherapy, the College of Occupational Therapy, the Society of British Neurological Surgeons, and the Society for Research in Rehabilitation.

43 NL Childs, WN Mercer, and HW Childs, ‘Accuracy of Diagnosis of Persistent Vegetative State’ (1993) 43 Neurology 1465.

44 K Andrews and others, ‘Misdiagnosis of the Vegetative State: Retrospective Study in a Rehabilitation Unit’ (1996) 313 BMJ 13.

45 C Schnakers and others, ‘Diagnostic Accuracy of the Vegetative and Minimally Conscious State: Clinical Consensus Versus Standardized Neurobehavioural Assessment’ (2009) 9 BMC Neurology 35.

46 See, for example, C Borthwick ‘The Permanent Vegetative State: Ethical Crux, Medical Fiction?’ (1996) 12 Issues L. & Med. 167; A Bisset-Johnson and P Ferguson, ‘Striving to Keep Alive? Care and Treatment Decisions Affecting Severely Handicapped Patients in Britain’ (1997) 4 EJHL 321; J Keown, ‘The Legal Revolution: From “Sanctity of Life” to “Quality of Life” and “Autonomy”’ (1998) 14 J. Contemp. Health L. & Pol'y 253; R Huxtable ‘Withholding and Withdrawing Nutrition/Hydration: The Continuing (Mis)Adventures of the Law’ (1999) 21 JSWFL 339.

47 RCP (n 28).

48 The criterion for inclusion in the study was that the brain injury was more than 1-month old.

49 See D Wade ‘Misdiagnosing the Persistent Vegetative State’ (1996) 313 BMJ 943.

50 In relation to one of the ‘misdiagnosed’ patients, Andrews *et al.* were not granted permission to report data.

51 See also Wade (n 49).

52 The court has also made this a requirement of diagnostic procedure prior to seeking declaratory relief: *W v M* [2011] EWHC 2443 (Fam).

53 RCP (n 28) 25.

54 For an extended discussion of what the authors term a ‘diagnostic illusory’, see S Nettleton, J Kitzinger, and C Kitzinger, ‘A Diagnostic Illusory? The Case of Distinguishing Between “Vegetative” and “Minimally Conscious” States' (2014) 116 Soc. Sci. Med. 134.

55 RCP (n 28) 14.

56 Wade (n 49); E Racine and others, ‘Observations on the Social and Ethical Aspects of Disorders of Consciousness' (2010) 37 Can. J. Neurol. Sci. 758.

57 J Howe, ‘The Persistent Vegetative State, Treatment Withdrawal and the Hillsborough Disaster: Airedale NHS Trust v Bland’ (2006) 6 Pract. Neurol. 238.

58 For a more sceptical reading of the *Bland* case in this respect, see J Bridgeman ‘Declared Innocent?’ (1995) 3 Med. L. Rev. 117.

59 See A Grubb ‘R v Bingley Magistrates' Court, ex parte Morrow’ (1995) 3 Med. L. Rev. 86 for a discussion of the unsuccessful attempt by Rev. Morrow to require the Bingley Magistrates to issue a summons against Bland's doctors for murder.

60 *Aintree University Hospitals NHS Foundation Trust v James* [2013] UKSC 67, para. 22.

61 See, for example, DB Wexler and BJ Winick, *Essays in Therapeutic Jurisprudence* (Caroline University Press, Durham 1991); DB Wexler, ‘Therapeutic Jurisprudence: An Overview’ (2000) 17 T. M. Cooley L. Rev. 125.

62 *Airedale NHS Trust v Bland* [1993] AC 789, 816.

63 Ibid, 863.

64 S Holland, C Kitzinger, and J Kitzinger, ‘Death, Treatment Decisions and the Vegetative State: Evidence from Families and Experts' (2014) 17 Med. Health Care Philos. 413.

65 *Re C* [2010] EWHC 3448 (FAM).

66 *NHS Trust v AW* [2013] EWHC 78 (COP).

67 *NHS Trust v I* [2003] EWHC 2243 (Fam).

68 *Re JD* [2012] EWHC 4420 (Fam).

69 *NHS Trust v I* [2003] EWHC 2243 (Fam).

70 *NHS Trust v AW* [2013] EWHC 78 (COP).

71 *Trust A v M* [2005] EWHC 807 (Fam).

72 *Re C* [2010] EWHC 3448 (FAM).

73 <http://www.healthtalk.org/peoples-experiences/nerves-brain/family-experiences-vegetative-and-minimally-conscious-states/family-experiences-applying-court-treatment-withdrawal> (last accessed 29 January 2015).

74 C Kitzinger and J Kitzinger, ‘Withdrawing Artificial Nutrition and Hydration from Minimally Conscious and Vegetative Patients: Family Perspectives' (2014) J. Med. Ethics, doi:10.1136/medethics-2013-101799.

75 Ibid.

76 Quoted in S Halliday, C Kitzinger, and J Kitzinger, ‘Law in Everyday Life and Death: A Socio-Legal Study of Chronic Disorders of Consciousness' (2014) 35 LS 55, at p. 65.

77 On legal fictions, see LL Fuller, *Legal Fictions* (Stanford University Press: Stanford 1967).

78 R Blendon, J Benson, and M Hermann, ‘The American Public and the Terri Schiavo Case’ (2005) 165 Arch. Intern. Med. 2580.

79 See, eg, HL Deb 10 January 2005, vol. 668 cc11-26; HL Deb 15 March 2005, vol. 670 cc1275-324; HL Deb 17 March 2005, vol. 670 cc1441-76.

80 Joint Committee Report on the Draft Mental Incapacity Bill, Session 2002-3, vol. 1 (HL Paper 189-1, HC 1083-1), para. 129.

81 See R Barry, ‘The Papal Allocution on Caring for Persons in a "Vegetative State"’ (2004) 20 Issues L. & Med. 155.

82 *W v M* [2011] EWHC 2443 (Fam), para. 116.

83 See Kitzinger and Kitzinger (n 74).

84 <http://www.healthtalk.org/peoples-experiences/nerves-brain/family-experiences-vegetative-and-minimally-conscious-states/artificial-nutrition-and-hydration/> (last accessed 29 January 2015).

85 RCP (n 28) 61.

86 Via the grant of a Health and Welfare Lasting Power of Attorney: Mental Capacity Act 2005, s. 24.

87 Ibid, ss. 9–11. Note, however, that, under s. 11(8) an attorney can only refuse life-sustaining treatment if the grant of the power of attorney expressly provides for this.

88 Currently only 4% of the population of England and Wales report having made an Advance Decision and only 4% report having appointed anyone as their Health and Welfare Lasting Power of Attorney (YouGov 2013; <http://www.compassionindying.org.uk/knowledge-end-life-rights-and-choices-yougov-poll2013>) (last accessed 29 January 2015).

89 Jennett (n 17).

90 L Willmott, BP White, and J Downie, ‘Withholding and Withdrawal of “Futile” Life-Sustaining Treatment: Unilateral Medical Decision-Making in Australia and New Zealand’ (2013) 20 JLM 907.

91 S Gevers, ‘Withdrawing Life Support from Patients in a Persistent Vegetative State: The Law in the Netherlands' (2005) 11 EJHL 347; I de Beaufort, ‘Patients in a Persistent Vegetative State – A Dutch Perspective’ (2005) 352 N. Engl. J. Med. 2373.

92 MN Slabbert, *Medical Law in South Africa* (Wolters Kluwer: The Netherlands 2011).

93 A Meisel and KL Cerminara, *Right to Die: The Law of End-of-Life Decisionmaking* (Aspen Publishers: New York, 3rd edn, 2004); S Rode ‘End-of-Life Decisionmaking for Patients in Persistent Vegetative States: A Comparative Analysis' (2007) 30 Hastings Int'l & Comp Law Rev. 477.

94 Willmott, White, and Downie (n 90) 907.

95 D Morgan, *Issues in Medical Law and Ethics* (Cavendish: London 2001), 90.

96 A judgment of the French Conseil d'Etat that a doctor's decision to withdraw CANH from a vegetative patient was lawful is currently being considered by the Grand Chamber of the European Court of Human Rights (*Lambert and others v France*: application no. 46043/14). The applicants claim, *inter alia*, that the withdrawal of treatment would constitute a breach of Articles 2 and 3 of the ECHR. There was a hearing of the Grand Chamber on 7 January 2015. The judgment will be issued in due course.

97 See FG Pizzetti, *Alle Frontiere della Vita: Il Testamento Biologico Tra Valori Costituzionali e Promozione della Persona* (Giuffrè: Milano 2008); C Casonato, *Introduzione al Biodiritto* (G. Giappichelli: Torino 2009); Pioggia, ‘Consenso informato e rifiuto di cure: dal riconoscimento alla soddisfazione del diritto’ (2009) 3 Giornale di Diritto Amministrativo 21.

98 1996 SC 301.

99 Ibid, 319.

100 *Death and the Procurator Fiscal* (Crown Office and Procurator Fiscal Service, 2008), para. 17.3.

101 See, for example, JK Mason and GT Laurie, *Mason and McCall Smith's Law and Medical Ethics* (Oxford University Press: Oxford, 9th edn, 2013).

102 *Re T* (*Adult: Refusal of Medical Treatment*) [1992] 4 All ER 645; *Airedale NHS Trust v Bland* [1993] AC 789; *Chester v Afshar* [2004] UKHL, 4.

103 The exception to this general principle, of course, is where necessity demands treatment despite the absence of consent (*Re F* [1990] 2 AC 1). Such has been codified in relation to incapax patients by s. 5 of the Mental Capacity Act 2005.

104 [2013] UKSC 67.

105 Per Lady Hale, para. 22.

106 *Airedale NHS Trust v Bland* [1993] AC 789.

107 For a discussion of delays caused by litigation in the context of the best interests of infants, see G Birchley and R Huxtable, ‘Critical Decisions for Critically Ill Infants: Principles, Processes, Problems' in Fovargue and Mullock (n 24).

108 See also C Kitzinger and J Kitzinger, ‘Family Perspectives on Proper Medical Treatment’ in Fovargue and Mullock (n 24).

109 *Burke v General Medical Council* [2005] EWCA 1003.

110 Para. 6.18.

111 Practice Direction 9E, para. 5.

112 The Code of Practice provides that the following decisions should also be brought before a court: (1) cases involving organ or bone marrow donation by a person who lacks capacity to consent, (2) cases involving the proposed non-therapeutic sterilisation of a person who lacks capacity to consent to this (eg for contraceptive purposes), and (3) all other cases where there is a doubt or dispute about whether a particular treatment will be in a person's best interests (para. 8.18).

113 British Medical Association (n 16).

114 *Treatment and Care Towards the End of Life: Good Practice in Decision-Making* (General Medical Council, 2010), paras. 106 and 120.

115 Ibid, para. 121.

116 RCP (n 28).

117 See J Kitzinger and C Kitzinger, ‘The “Window of Opportunity” for Death After Severe Brain Injury: Family Experiences' (2012) 35 Sociol. Health Ill. 1095.

118 Per Lord Goff, at 873; per Lord Browne-Wilkinson at 885; see also *Burke v General Medical Council* [2005] EWCA 1003.

119 S.I. 2007/1744. The Court of Protection Rules 2007 are made under the authority of the Mental Capacity Act 2005, ss. 51 and 52.

120 See, for example, Lord Goff at 863.

121 Practice Direction 9E, para. 5 offers a list of potential decisions that are to be regarded as ‘serious medical treatment’ and that should be brought before the court for consideration. Whereas decisions to withdraw or withhold CANH from PVS and MCS patients are specifically mentioned, the withholding or withdrawing of other life-sustaining treatments (including antibiotics) is not. Yet, ‘serious medical treatment’ is defined as including: ‘providing, withdrawing or withholding treatment in circumstances where … the treatment, procedure or investigation proposed would be likely to involve serious consequences for [the patient].’ A decision to withhold antibiotics in the event of a life-threatening infection could, one might argue, fall within this definition. The Code of Practice of the Mental Capacity Act 2005, however, envisages that only CANH decisions must be brought to the court. See Sections 6.18 and 8.18.

122 RCP (n 28), Section 5.

123 [2010] EWHC 3448 (Fam), at para. 17.

124 See Kitzinger and Kitzinger (n 117).

125 Lewis (n 21).

126 This is discussed in greater depth in S Halliday, C Kitzinger, and J Kitzinger, ‘Law in Everyday Life and Death: A Socio-Legal Study of Chronic Disorders of Consciousness' (2014) 35 LS 55, 74.

127 Quoted in Halliday, Kitzinger, and Kitzinger (n 76), 71.

128 Howe (n 57).

129 C Kitzinger and J Kitzinger, (2015 in press) ‘Court Applications for Withdrawal of Clinically Assisted Nutrition and Hydration from Patients in a Permanent Vegetative State: Family Views and Experiences' J Med Ethics.

130 C Kitzinger and J Kitzinger, ‘Grief, Anger and Despair in Relatives of Seriously Brain-Injured Patients: Responding Without Pathologizing’ (2014) 28 Clin. Rehabil. 627.

131 Kitzinger and Kitzinger (n 117).

132 Kitzinger and Kitzinger (n 129).

133 Quoted in Halliday, Kitzinger, and Kitzinger (n 76), 66.

134 See, eg, D Wade, ‘Ethical Issues in Diagnosis and Management of Patients in the Permanent Vegetative State’ (2001) 322 BMJ 352; Racine and others (n 56).

135 *Airedale NHS Trust v Bland* [1993] AC 789, per Lord Hoffman in the Court of Appeal at 833; per Lord Wilkinson-Browne in the House of Lords at 880; per Lord Goff at 896.

136 Formby, Cookson, and Halliday (n 25).

137 WS van Erp and others, ‘The Vegetative State/Unresponsive Wakefulness Syndrome: A Systematic Review of Prevalence Studies’ (2014) 21 Eur. J. Neurol. 1361. The little evidence that exists suggests that prevalence varies within a range of 0.2–6.1 patients per 100,000 members of the population.

138 The problem of families funding declaratory relief proceedings is discussed in *W v M* [2011] EWHC 2443 (Fam).

139 NHS Confederation, ‘Key Statistics on the NHS' <http://www.nhsconfed.org/priorities/political-engagement/Pages/NHS-statistics.aspx#funding> (last accessed 29 January 2015).

140 <https://www.nice.org.uk/glossary?letter=q> (last accessed 29 January 2015). Quality of life is often measured in terms of the patient's ability to perform the activities of daily life, freedom from pain, and mental disturbance.

141 K Claxton and others, *Methods for the Estimation of the NICE Cost Effectiveness Threshold* (University of York, Centre for Health Economics: York, Research Paper 81, 2013). NICE itself applies a more conservative threshold of £20,000.

142 The decision in *W v M and others* [2011] EWHC 2443 (Fam) not to permit the withdrawal of CANH has been criticised by a number of scholars. See, for example, A Mullock, ‘Deciding the Fate of a Minimally Conscious Patient: An Unsatisfactory Balancing Act?’ (2012) 20 Med. L. Rev. 460; E Jackson, ‘The Minimally Conscious State and Treatment Withdrawal: *W v M*’ (2013) 39 J. Med. Ethics 17. We do not address this question in this article. Our position relates to the state of the law as it currently is.

143 Since the development of MCS as a diagnostic category in 2002, we know of this having happened only twice: *W v M and others* [2011] EWHC 2443 (Fam); and *NHS Trust v L* [2013] EWHC 4313 (Fam). It is possible, of course, that in other cases revisions of diagnosis caused by preparations for declaratory relief applications led to the abandonment of those proceedings.

144 For a consideration of this issue incorporating medical ethics, see C Constable, ‘Withdrawal of Artificial Nutrition and Hydration for Patients in a Permanent Vegetative State: Changing Tack’ (2012) 26 Bioethics 157.

145 *Airedale NHS Trust v Bland* [1993] AC 789, per Lord Keith at 859; Lord Goff at 863; Lord Lowry at 876.

146 B Markesinis and J Fedtke, *Judicial Recourse to Foreign Law: A New Source of Inspiration?* (Routledge: London, 2006).

147 RCP (n 28) 77. See also Derick Wade's commentary on the RCP guidelines: ‘Patients with Prolonged Disorders of Consciousness: More than a Clinical Challenge’ (2014) 14 Pract. Neurol. 2; and Kitzinger and Kitzinger (n 130).

148 [1993] 1 All ER 821 at 855, (1993) 12 BMLR 64 at 100.

149 R Dworkin, *Taking Rights Seriously* (Harvard University Press: Harvard 1977).

150 *W v M and others* [2011] EWHC 2443 (Fam).

151 L Zedner, ‘Securing Liberty in the Face of Terror: Reflections from Criminal Justice’ (2005) 32 J. Law & Soc. 507.

152 <http://www.nhsconfed.org/resources/key-statistics-on-the-nhs> (last accessed 29 January 2015).

153 See S Halliday, *Judicial Review and Compliance with Administrative Law* (Hart Publishing: Oxford 2004).

154 Whereas Parliament is well suited to the unenviable task of resolving difficult and consequential policy decisions.

155 For this reason, we respectfully disagree with Lewis when she suggests that the court is ‘rubber-stamping’ the decision of doctors. See Lewis (n 21).

156 See, for example, J Way, A Black, and JR Curtis, ‘Withholding Life Support and Resolution of Conflict with Families' (2002) 325 BMJ 1342.

157 Baroness Ashton, Parliamentary Under-Secretary, Department for Constitutional Affairs, HL Deb, 25 January 2005, c1240.

158 The Law Commission first published its report in 1995: *Mental Incapacity* (Law Commission Report 231, 1995). The Green Paper was published in 1997: *Making Decisions on behalf of Mentally Incapacitated Adults* (Green Paper, Cm 3803, 1997).

159 S.I. 2007/1744.

160 Sections 51 and 52.

161 Mental Capacity Act 2005, s. 43(2).

